# Supplementary material for: MetaRibo-Seq measures translation in microbiomes
Source: Nat Commun. 2020 Jun 29;11:3268. doi: 10.1038/s41467-020-17081-z (PMC7324362; doi:10.1038/s41467-020-17081-z)
Supplement: Supplementary file 10 — Supplementary Data 7 [file 41467_2020_17081_MOESM10_ESM.zip › File2/Confidence_VeryHigh_Taxonomy/155673_out.krona.html]

Javascript must be enabled to view this page.

members
magnitude
magnitudeUnassigned
count
unassigned
taxon
rank

155673\_out

27

superkingdom
27
2

27
1239
phylum

186801
27
class

27
186802
order

31979
23
family

1485
23
genus


SRS011134\_contig\_number\_contig-100\_359.301845SRS014855\_contig\_number\_15369SRS015578\_contig\_number\_22812SRS015579\_contig\_number\_1419SRS015694\_contig\_number\_18143SRS017821\_contig\_number\_14268SRS022071\_contig\_number\_36435SRS022609\_contig\_number\_47073SRS024075\_contig\_number\_12440SRS049900\_contig\_number\_contig-100\_1149.122167SRS052697\_contig\_number\_14831SRS075773\_contig\_number\_4790SRS098644\_contig\_number\_35220SRS1041033\_contig\_number\_contig-100\_1100.127779SRS1041038\_contig\_number\_14837SRS105153\_contig\_number\_34357SRS1055067\_contig\_number\_9484SRS143342\_contig\_number\_12416SRS143372\_contig\_number\_7264SRS143466\_contig\_number\_32450
20
1262828
species

species
2

SRS047014\_contig\_number\_25783SRS076756\_contig\_number\_19557
2293016

2293015
1

SRS144183\_contig\_number\_18779
species

186806
1
family

1
1730
genus

species

SRS015854\_contig\_number\_16967
1
1262895

1
186803
family

species
1

SRS077194\_contig\_number\_28965
1898203

species
2

SRS015217\_contig\_number\_23735SRS019161\_contig\_number\_41889
1676155
